# Tracheostomy in COVID-19: A retrospective cohort study of outcomes and mortality predictors in a specialized infectious disease hospital in Brazil

**DOI:** 10.1371/journal.pone.0326531

**Published:** 2025-06-18

**Authors:** André Figueiredo Accetta, Denise Machado Medeiros, Maria Pia Diniz Ribeiro, Sandra Wagner Cardoso, Isabel Cristina Ferreira Tavares, Valdilea Gonçalves Veloso, Hugo Boechat Andrade, André Miguel Japiassú

**Affiliations:** 1 School of Medicine, Universidade Federal Fluminense (UFF), Niterói, Rio de Janeiro, Brazil; 2 Instituto Nacional de Infectologia Evandro Chagas (INI), Fundação Oswaldo Cruz (Fiocruz), Rio de Janeiro, Rio de Janeiro, Brazil; 3 Instituto Biomédico, Universidade Federal Fluminense (UFF), Niterói, Rio de Janeiro, Brazil; University of Sao Paulo: Universidade de Sao Paulo, BRAZIL

## Abstract

**Background:**

The COVID-19 pandemic profoundly impacted critical care practices, leading to a significantly increase in tracheostomies for patients with respiratory failure. This study aimed to investigate prognostic factors associated with mortality in COVID-19 patients undergoing tracheostomy for respiratory failure in the intensive care unit of a specialized infectious disease hospital in Brazil.

**Research Question:**

What is the impact of the timing of tracheostomy on the outcome of COVID-19 patients?.

**Materials and methods:**

This retrospective cohort study analyzed 356 COVID-19 patients who underwent tracheostomy at the Evandro Chagas National Institute of Infectious Diseases (INI) in Brazil between May 2020 and December 2023. Data on demographics, comorbidities, illness severity, surgical factors, and outcomes were extracted from medical records. Multivariate logistic regression was performed to identify factors associated with hospital mortality.

**Results:**

The overall hospital mortality rate was 68%. Independent factors associated with mortality included age over 60 years, chronic pulmonary disease, diabetes, lack of COVID-19 vaccination, hemodialysis on the day of surgery, and a PaO_2_/FiO_2_ ratio lower than 200 on the day of surgery. The timing of tracheostomy (after 21 days of mechanical ventilation) was not associated with mortality.

**Conclusions:**

This study offers valuable insights into the complex factors influencing mortality in critically ill COVID-19 patients. Our findings underscore the importance of assessing both patient characteristics and illness at the time of tracheostomy to predict outcomes which is more critical than basing the decision solely on the duration of endotracheal intubation or mechanical ventilation.

## Introduction

Coronavirus disease 2019 (COVID-19) pandemic caused by SARS-CoV-2, has transformed the landscape of medical practice across all specialties, particularly in emergency departments and intensive care units. It has led to an unprecedented surge in critically ill patients [1,2]. This has required greater expertise from multidisciplinary teams in managing mechanical ventilation for the treatment of pneumonitis. Orotracheal intubation is often necessary for ventilating these patients, and in most cases, the duration of invasive ventilation is longer than for other viral pneumonias [3].

Tracheostomy is a well-established surgical procedure in ICUs, primarily indicated for facilitating long-term ventilation [2]. Other indications include managing upper airway obstruction, improving ventilation comfort, and protection against aspiration [4].

Before COVID-19 pandemic, approximately 10% of ICU patients requiring advanced respiratory support underwent tracheostomy [5]. However, during the pandemic, the need for tracheostomy fluctuated significantly, with rates ranging from 15% to 60%, considerably higher than pre-pandemic levels [2,6].

Tracheostomy benefits patients requiring prolonged ventilation by allowing for reduced or discontinued sedation and removal of endotracheal tube, thereby preventing complications associated with prolonged intubation [7,8]. It may also shorten ICU stays, thus increasing bed availability [6,9].

Prolonged orotracheal intubation combined with the use of corticosteroids and neuromuscular blockers contributes to the weakening of respiratory muscles, complicating the extubation process and the return to independent breathing [5].

Given that COVID-19 patients typically require longer periods of ventilation than those with other viral pneumonias, it is not surprising that studies have shown tracheostomy in this population can confer a survival benefit, aiding in gradual withdrawal of ventilatory support [2,10].

Tracheostomy and its subsequent management require a trained team to minimize complications and ensure patient safety incidents. In situations of a staff shortages, tracheostomy can reduce resources needed for managing patients on mechanical ventilation, alleviating critical care burden [11].

As an aerosol-generating procedure, tracheostomy increases the risk of viral exposure for healthcare teams [12]. Due to COVID-19’s high transmissibility and risk of severe disease, it is essential to carefully weigh the benefits for patients against potential risks to healthcare workers. Proper team training and the use of appropriate personal protective equipment are crucial for minimizing risks. Additionally, complications related to procedure, such as hemorrhage and tracheal stenosis, should also be considered [13].

In light of above, it is important to study this procedure in greater depth, using a larger sample and comparing the results and outcomes with current medical literature, particularly among patients in this specific epidemiological context.

The primary objective of this study is investigating prognostic factors associated with the outcomes of tracheostomies performed during COVID-19 pandemic.

## Materials and methods

This study is a retrospective and observational cohort study of all patients with confirmed COVID-19 diagnosis who underwent tracheostomy, by the same surgeon, utilizing the same surgical technique, at the Intensive Care Unit (ICU) of the Evandro Chagas National Institute of Infectious Diseases (INI) in Rio de Janeiro, Brazil, between May 2020 and December 2023. The INI is a specialized infectious disease hospital and has served as a reference center for COVID-19 hospitalization since the beginning of the pandemic. This study was reported in accordance with the Strengthening the Reporting of Observational Studies in Epidemiology (STROBE) guidelines for observational studies, and was approved by Research Ethics Committee (CAAE: 66495523.0.0000.5262). Access to the medical records occurred between January 2, 2024, and March 2, 2024. The authors had access to all medical records, but only for research purposes. During the data collection process, personal information of the participants that was not necessary for the study was kept confidential and anonymized, in accordance with ethical and privacy standards. No information that could directly identify the participants was used or disclosed.

### Patient population

The analysis included hospitalized adult patients (aged 18 or older) with confirmed COVID-19, diagnosed by positive RT-PCR from a nasopharyngeal swab or tracheal aspirate. Patients also had respiratory failure requiring mechanical ventilation and had underwent tracheostomy for respiratory management. Exclusion criteria included tracheostomy performed for non-respiratory reasons or incomplete medical records.

### Surgery

All tracheostomies were open and performed using the same surgical technique. All procedures were carried out by the same surgeon at the bedside in isolation units of the ICU, in individual rooms with negative pressure, with the patient sedated.

### Data collection

Data were retrospectively collected from patients’ medical records using a standardized data extraction form. The following baseline data were collected:

- **Demographics:** Age, sex at birth, body mass index (BMI), COVID-19 vaccination status (number of doses). All patients operated before 1^st^ February 2021, when the vaccine was introduced, were considered unvaccinated.
- **Comorbidities:** Diabetes, cardiovascular disease, chronic pulmonary disease, hemodialysis (previous), HIV/AIDS, hypertension, neoplastic disease.
- **Clinical Characteristics:**
  - **Before tracheostomy:** Time of COVID-19 evolution (days), Simplified Acute Physiology Score 3 (SAPS 3) at ICU admission, Sequential Organ Failure Assessment (SOFA) score at ICU admission, reason for ICU admission, duration of mechanical ventilation (days)
  - **On the day of tracheostomy:** Ratio of arterial oxygen partial pressure to fractional inspired oxygen (PaO_2_/FiO_2_ or P/F in mmHg), use of vasopressors, presence of coagulation disorders, and ongoing hemodialysis.

Coagulation disorders were defined as thrombocytopenia (<100,000), prolonged Prothrombin Time (PT) and/or activated Partial Thromboplastin Time (aPTT), active bleeding and/or the need for plasma and/or platelet transfusions for procedure.

- **During tracheostomy:** Duration of procedure (minutes), complications, technical difficulties;

- **Post-tracheostomy outcomes:** Primary: In-hospital mortality; Secondary: ICU mortality, postoperative complications, duration of mechanical ventilation (days), duration of tracheostomy (days until decannulation), decannulation status (yes/no), length of hospital stay, and length of ICU stay.

### Statistical analysis

All statistical analyses were conducted using the R® application for Microsoft Windows®. A p-value of less than 0.05 (95% confidence interval – CI) was considered statistically significant. A minimum sample size of 323 patients was calculated to ensure a 95% confidence interval with a 5% margin of error for a population with an estimated in-hospital mortality rate of 70%.

Descriptive statistics were employed to summarize patient characteristics and clinical data. Continuous variables were presented as medians (interquartile range, IQR), while categorical variables were reported as absolute frequencies (N) and proportions (%). For some continuous variables with a median of 0, both mean and standard deviation were also provided. Comparisons between groups were performed using the Wilcoxon test or Kruskal-Wallis tests for continuous variables and the chi-squared (χ²) Pearson test for categorical variables.

Variables associated with the primary outcome were included in a multivariate logistic regression analysis to identify factors independently associated with in-hospital mortality, while adjusting for potential confounders. To reduce collinearity, variables correlated with the outcome in the final model were selected based on the Variance Inflation Factor (VIF) [14]. Continuous variables were categorized using the Youden index cut-off point to minimize the influence of outliers on outcome estimation.

## Results

Out of 5,443 patients hospitalized with Acute Respiratory Distress Syndrome (ARDS)/COVID-19, 3,755 (69%) were admitted to ICU. A total of 356 (9.5%) patients who underwent tracheostomy for COVID-19-related respiratory failure were included in analysis, none were excluded from study. Of these, 114 (32%) survived and were followed until discharged, while 242 (68%) died. Three patients required emergency tracheostomy due to failed intubation.

Demographic and clinical characteristics are summarized in **Table 1**. The median patient age was 63 years (IQR: 52–72), with 59.6% of the cohort being male. Patients who died were significantly older than survivors (median age 66 vs. 55 years, p < 0.01). Significant differences were also observed in prevalence of diabetes (31.4% vs. 17.5%, p = 0.01), cardiovascular disease (0.1% vs. 0%, p = 0.04), and chronic pneumopathy (15.3% vs. 6.1%, p = 0.01) between the two groups.

**Table 1:** COVID-19 tracheostomy – Demographics and clinical characteristics.

	ALL	ALIVE	DEAD	Test statistic
Proportion or median	N = 356 (100%)	N = 114 (32%)	N = 242 (68%)	
Age years (median, interquatile range)	63 (52–72)	55 (44–66)	66 (55–74)	p < 0.01^2^
Body mass index				
*Median, interquartile range*	28 (25–33)	29 (25.9–34.1)	28 (25–32)	p = 0.01^2^
*>30 (%)*	137 (38.4)	54 (47.3)	83 (34.2)	p = 0.02^1^
Cardiovascular disease	14 (3.9)	1 (0.8)	13 (5.3)	p = 0.04^1^
Congestive heart disease	12 (3.3)	3 (2.6)	9 (3.7)	p = 0.60^1^
COVID-19 evolution, days (median, interquatile range)	7 (4–9)	7 (4–10)	6 (3.0–8.1)	p = 0.09^2^
Chronic pneumopathy	44 (12.3)	7 (6.1)	37 (15.2)	p = 0.01^1^
Diabetes	96 (26.9)	20 (17.5)	76 (31.4)	p = 0.01^1^
Hemodialysis (previous)	4 (1.1)	1 (0.8)	3 (1.2)	p = 0.76^1^
HIV/AIDS	22 (6.1)	8 (7.0)	14 (5.7)	p = 0.65^1^
Hypertension	213 (59.8)	62 (54.3)	151 (62.3)	p = 0.15^1^
Neoplastic disease	7 (1.9)	1 (0.8)	6 (2.4)	p = 0.31^1^
Sex at birth (male)	212 (59.5)	74 (64.9)	138 (57.0)	p = 0.16^1^
Oxygen delivery/therapy device at arrival				p = 0.06^1^
*Room air*	44 (12.3)	15 (13.1)	29 (11.9)	
*Conventional oxygen therapy*	206 (57.8)	69 (60,5)	137 (56.6)	
*Invasive endotracheal intubation*	106 (29.7)	30 (26.3)	76 (31.4)	
Reason for ICU admission				p = 0.34^1^
*Coma*	5 (1.4)	1 (0.8)	4 (1.65)	
*Respiratory failure*	339 (95.2)	107 (93.8)	232 (95.8)	
*Shock/ septic shock*	12 (3.3)	6 (5.2)	6 (2.47)	
SAPS, points (median, interquatile range)	53 (44.0–67.2)	48 (43.0–63.1)	4 (46–70)	p < 0.01^2^
SOFA, points (median, interquatile range)	4 (2–7)	3 (2–7)	4 (2–8)	p = 0.07^2^
Time of disease at admission, days (median, interquatile range)	7 (4–9)	7 (4–10)	6 (3.0–8.1)	p = 0.09^2^
Vaccines doses (COVID-19, any)	305^a^ (85.6)			p = 0.01^1^
*0*	228 (64.0)	86 (75.4)	142 (58.6)	
*1*	23 (6.4)	2 (1.7)	21 (8.6)	
*≥2*	54 (15.1)	13 (11.4)	41 (16.9)	

^1^Pearson.

^2^Wilcoxon.

^a^Vaccination status information after 1 Feb 2021. COVID-19: Coronavirus disease 2019 by SARS-CoV-2. Cardiovascular disease: 11 cases of myocardial infarction, 2 cases of cerebrovascular disease and 1 case of peripheral arterial disease. Congestive heart disease: heart functional classification by New York Heart Association – NYHA ≥II. Chronic pneumopathy: GOLD III/IV chronic obstructive pulmonary disease. Hemodialysis (previous): end-stage renal disease on hemodialysis before admission; HIV/AIDS: human immunodeficiency virus/ acquired immunodeficiency syndrome; Neoplastic disease: 5 cases of solid tumors and 2 cases of hematologic neoplasms undergoing oncological treatment.; SAPS (Simplified Acute Physiology Score) 3. SOFA: sequential organ failure assessment score.

Most patients were already intubated upon ICU admission. The median duration of illness at admission was 7 days (IQR: 4–9), and they had been hospitalized at other institutions for a median of 2 days prior to being transferred to our hospital.

Seventy-seven patients (21.6%) received at least one dose of COVID-19 vaccine, and there was a significantly higher percentage of patients who had not received any vaccination among those who died (69.6% vs. 30.3%, p = 0.01).

Patients who died were not only more likely to have pre-existing comorbidities but also presented with higher severity of illness at tracheostomy. Patients who died did not show a significant difference in the time of disease at admission compared to survivors (6.0 vs. 7.0 days, p = 0.09).

Table 2 examines characteristics of patients who received mechanical ventilation for less than or more than 2 weeks. The median duration of mechanical ventilation prior to tracheostomy was 16.0 days (IQR: 14.0–19.0) for all patients. Those who underwent tracheostomy after more than 14 days of mechanical ventilation had higher SAPS 3 scores (median 54.0 vs. 50.2, p = 0.01) and higher SOFA scores (median 4.0 vs. 3.0, p = 0.01) at ICU admission. These patients also experienced a longer length of stay in the ICU (median 35.0 vs. 29.0 days, p = 0.01) and a longer total hospitalization length (median 40.0 vs. 33.0 days, p < 0.01), though hospital mortality rates were similar (67.8% vs. 68.2%, p = 0.95).

**Table 2:** COVID-19 tracheostomy – Characteristics of patients on mechanical ventilation for less than and more than 14 days.

	<14 days	>14 days	Test Statistic
Proportion or median – N = 356 (100%)	N = 107 (30%)	N = 249 (70%)	
Age years (median, interquatile range)	62.2 (47.2–73.8)	63.6 (52.9–71.2)	p = 0.55^2^
BMI (interquatile range)	27.5 (24.6–31.1)	27.9 (24.9–33.3)	p = 0.08^2^
Complication during surgery	7 (6.5)	34 (13.6)	p = 0.05^1^
Decannulated patients	31 (28.9)	74 (29.7)	p = 0.89^1^
Hospital mortality	73 (68.2)	169 (67.8)	p = 0.95^1^
ICU mortality	69 (64.4)	165 (66.2)	p = 0.75^1^
Duration of MV before TCT, days (median, interquatile range)	13 (12–14)	18 (16–20)	p < 0.01^2^
Duration of TCT, days (median, interquatile range)	17 (10.0–35.8)	21 (8–42)	p = 0.38^2^
LOS before INI, days (median, interquatile range)	2 (0–3)	2 (1–4)	p = 0.70^2^
LOS INI, days (median, interquatile range)	33 (24.0–50.8)	40 (27–60.7)	p < 0.01^2^
LOS INI before ICU, days (median, interquatile range)	0.0 (0.0–0.0)	0.0 (0.0–0.0)	p = 0.11^2^
LOS INI ICU (days, median, interquatile range)	29 (23–40.5)	35 (27–52.0)	p < 0.01^2^
P/F on surgery day (interquatile range)	254 (181.5–339.5)	265 (190–400)	p = 0.11^2^
Postoperative complications	8 (7.4)	17 (6.8)	p = 0.83^1^
SAPS 3, points (median, interquatile range)	50 (42.2–59.8)	54 (46–69)	p = 0.01^2^
Sex at birth (male)	61 (57.0)	151 (60.6)	p = 0.52^1^
SOFA, points (median, interquatile range)	3 (2–6)	4 (2–8)	p = 0.01^2^
Weaning from MV	36 (33.6)	73 (29.3)	p = 0.42^1^

^1^Pearson.

^2^Wilcoxon. BMI: body mass index; ICU: intensive care unit; INI: Instituto Nacional de Infectologia; COVID-19: Coronavirus disease 2019 by SARS-CoV-2; LOS: length of stay; MV: mechanical ventilation; TCT: tracheostomy. P/F: the ratio of arterial oxygen partial pressure to fractional inspired oxygen; SAPS (Simplified Acute Physiology Score) 3. SOFA: sequential organ failure assessment score.

Table 3 details severity of illness at tracheostomy time and associated surgical complications. Hemodialysis on the day of surgery was significantly more common in the deceased group (40.0% vs. 21.0%, p < 0.01), and the P/F ratio on the day of surgery was notably lower in the deceased group (median 240.5 vs. 306.5, p < 0.01). However, there were no significant differences in postoperative complications (5.7% vs. 9.6%, p = 0.18) or technical difficulties during tracheostomy (10.3% vs. 14.0%, p = 0.31) between the deceased and surviving groups.

**Table 3:** COVID-19 tracheostomy – Severity of illness at tracheostomy time and surgical complications.

	ALL	ALIVE	DEAD	Test statistic
Proportion or median	N = 356 (100%)	N = 114 (32%)	N = 242 (68%)	
Amine use on surgery day	203 (57)	66 (5.7)	137 (56.6)	p = 0.82^1^
Coagulation issue on surgery day^a^	33 (9.2)	4 (3.5)	29 (11.9)	p = 0.01^1^
Hemodialysis on surgery day	121 (33.9)	24 (21.0)	97 (40.0)	p < 0.01^1^
P/F on surgery day (interquatile range)	261.5 (187.3–386.0)	306.5 (221.9–414.5)	240.5 (167– 62.1)	p < 0.01^2^
Duration of MV before TCT, days (median, interquatile range)	16 (14–19)	17 (14–19)	16 (14–19)	p = 0.68^2^
Postoperative complications	Yes – 25 (7.1)	11 (9.6)	14 (5.7)	p = 0.18^1^
*Accidental decannulation*	7 (1.9)	1 (0.8)	6 (2.4)	
*Hemorrhage*	9 (2.5)	3 (2.6)	6 (2.4)	
*Others*	5 (1.4)	3 (2.6)	2 (0.8)	
*Stenosis*	4 (1.1)	4 (3.5)	0 (0.0)	
	No – 331 (92.9)	103 (90.3)	228 (94.2)	
Surgical duration ≥40 min	20 (5.6)	8 (7.0)	12 (4.9)	p = 0.43^1^
Technical difficulties during surgery	Yes – 41 (11.5)	16 (14.0)	25 (10.3)	p = 0.31^1^
*Anatomic variations*	16 (4.4)	6 (5.2)	10 (4.1)	
*Adhesions*	6 (1.6)	3 (2.6)	3 (1.2)	
*False surgical route*	2 (0.5)	1 (0.8)	1 (0.4)	
*Hemorrhage*	5 (1.4)	2 (1.7)	3 (1.2)	
*Peritracheitis*	1 (0.2)	0 (0.0)	1 (0.4)	
*Stenosis*	1 (0.2)	1 (0.8)	0 (0.0)	
*Thyroid (enlarged)*	6 (1.6)	3 (2.6)	3 (1.2)	
*Ventilatory problem*	4 (1.1)	0 (0.0)	4 (1.6)	
	No – 315 (88.5)	98 (86.0)	217 (89.7)	

^1^Pearson.

^2^Wilcoxon. COVID-19: Coronavirus disease 2019 by SARS-CoV-2; MV: mechanical ventilation; P/F: the ratio of arterial oxygen partial pressure to fractional inspired oxygen; STE: standard error; TCT: tracheostomy.

^a^Coagulation issue was defined as: thrombocytopenia (<100,000), or prolonged Prothrombin Time (PT) and/or activated Partial Thromboplastin Time (aPTT); or active bleeding and/or the need for plasma and/or platelet transfusions for the surgical procedure.

Table 4 presents the outcomes. The median length of hospitalization for the entire cohort was 14.0 days (IQR: 16.0–19.0). Survivors had a significantly higher rate of decannulation (90.0% vs. 0.0%, p < 0.01) and a longer median duration of tracheostomy (39.0 vs. 12.0 days, p < 0.01). Additionally, survivors had a significantly longer median length of stay at hospital (55.0 vs. 30.5 days, p < 0.01) and a longer median length of stay in ICU specifically (41.5 vs. 30.0 days, p < 0.01).

**Table 4:** COVID-19 tracheostomy – Outcomes.

	ALL	ALIVE	DEAD	Test statistic
Proportion or median	N = 356 (100%)	N = 114 (32%)	N = 242 (68%)	
Decannulated patients	105 (29.5)	102 (89.4)	3 (1.2)	p < 0.01^1^
Duration of TCT, days (median, interquatile range)	14 (16–19)	39 (26–58.1)	12 (5.9–24.1)	p < 0.01^2^
Duration of MV, days (median, interquatile range)	30 (23–42)	30.5 (23–43)	29 (23–41)	p = 0.56^2^
LOS INI ICU, days (median, interquatile range)	33 (25–49)	41.5 (32–57.1)	30 (23–42)	p < 0.01^2^
LOS INI, days (median, interquatile range)	38 (26–58)	55 (42.0–80.1)	30.5 (23–42.1)	p < 0.01^2^
Weaning from MV	109 (30.6)	100 (87.7)	9 (3.7)	p < 0.01^1^
Mortality ICU	234 (65.7)	0 (0.0)	234 (96.6)	p < 0.01^1^

^1^Pearson.

^2^Wilcoxon. COVID-19: Coronavirus disease 2019 by SARS-CoV-2; ICU: intensive care unit; INI: Instituto Nacional de Infectologia; LOS: length of stay; MV: mechanical ventilation; TCT: tracheostomy.

Table 5 presents results of logistic regression analysis for in-hospital mortality. Multivariate analysis identified several independent predictors of in-hospital mortality: age over 60 years (OR 2.72, 95% CI: 1.42–5.20, p < 0.01), chronic pulmonary disease (OR 6.36, 95% CI: 1.81–22.33, p < 0.01), diabetes (OR 2.90, 95% CI: 1.40–6.03, p < 0.01), lack of COVID-19 vaccination (OR 1.53, 95% CI: 1.06–1.76, p = 0.03), hemodialysis on surgery day (OR 4.11, 95% CI: 2.04–8.30, p < 0.01), and a P/F ratio < 200 on surgery day (OR 2.44, 95% CI: 1.15–5.15, p = 0.02).

**Table 5:** COVID-19 tracheostomy – Multiple logistic regression – outcome: Hospital death.

FACTOR	OR	CI 95%	p-value
Age > 60 years-old	2.72	1.42 to 5.20	**<0.01**
Chronic pneumopathy	6.36	1.81 to 22.33	**<0.01**
Diabetes	2.90	1.40 to 6.03	**<0.01**
Duration of MV before TCT ≥ 21 days	0.94	0.40 to 2.23	0.89
Sex at birth (male)	0.95	0.48 to 1.91	0.88
Vaccination (No)	1.53	1.06 to 1.76	**0.03**
Surgery			
Hemodialysis on surgery day	4.11	2.04 to 8.30	**<0.01**
P/F ratio < 200 on surgery day	2.44	1.15. to 5.15	**0.02**
Postoperative complications	0.41	0.11 to 1.52	0.18
Technical difficulties	0.64	0.27 to 1.54	0.40

Bold: p-value <0.05. BMI: body mass index; CI: confidence interval; COVID-19: Coronavirus disease 2019 by SARS-CoV-2; Chronic pneumopathy: GOLD III/IV chronic obstructive pulmonary disease; MV: mechanical ventilation; OR: odds ratio; P/F: the ratio of arterial oxygen partial pressure to fractional inspired oxygen; TCT: tracheostomy.

## Discussion

This retrospective cohort study highlights the complex interplay of factors influencing in-hospital mortality in critically ill COVID-19 patients requiring tracheostomy for respiratory failure. Our analysis underscores the multifaceted challenges in managing this population, emphasizing the importance of early recognition, timely intervention, and a multidisciplinary approach to care.

The increased mortality risk associated with older age, comorbidities such as chronic pulmonary disease, diabetes, and a lack of COVID-19 vaccination highlights the need of a comprehensive assessment of patient vulnerability. Additionally, patients with lower P/F ratios on surgery day, indicating poorer oxygenation, further emphasize the necessity of prompt, intensive management to optimize outcomes. Identifying and addressing these risk factors early can contribute to improved patient prognoses.

A prospective multicenter study conducted in Brazil previously identified the SAPS-3 score as an independent predictor of in-hospital mortality [15]. Our study also reinforces the crucial role of a patient’s overall severity of illness at the time of tracheostomy in determining outcome. Patients presenting with higher severity of illness, evidenced by elevated SAPS 3 score, had significantly higher mortality rates. This finding underscores the need for a careful assessment of the overall clinical status before considering tracheostomy mortality.

Our study highlights the importance of timely tracheostomy in reducing complications associated with prolonged mechanical ventilation and improving outcomes. Although tracheostomy itself did not directly contribute to mortality in this study, the finding that patients remained critically ill at the time of procedure suggests that delaying tracheostomy may avoid unnecessary surgeries in cases where the intervention would not significantly impact outcomes.

While tracheostomy offers potential benefits for patients requiring prolonged mechanical ventilation, including reduced sedation needs, shorter weaning time, and decreased hospital and ICU stays [3], the optimal timing and patient selection for tracheostomy in COVID-19 remains controversial. This study, which spans three years of data collection, contributes to debate.

The timing of tracheostomy has long been a subject of discussion, since before the pandemic. A review by Andriolo [16] did not identify differences between early and late tracheostomy. Another meta-analysis of randomized controlled trials could not demonstrate significant benefits of tracheostomy within 10 days of intubation [17]. However, other studies have shown potential benefits of early tracheostomy in non-COVID-19 patients [18,19]. Puali [8] concluded that there was a correlation between a shorter intubation time before tracheostomy and the duration of mechanical ventilation and ICU stay. These results were similar to Shreckengost [20] with COVID-19 patients’ series, who also used the 14th day to classify tracheostomy as early or late.

At onset of pandemic, concerns emerged about potential aerosolization of viral particles during tracheostomy, which could increase risk of infection among healthcare workers [12,21,22]. Although some protocols [23–26] recommended obtaining a negative COVID-19 test prior to tracheostomy, this was not universally implemented, including in our study. At our institution, single-occupancy negative-pressure rooms and stringent use of personal protective equipment ensured staff safety. In addition, continuous training programs are in place. Consequently, the formal indications for tracheostomy, as established in the literature, were adhered to. Like other authors, we believe that a positive COVID-19 status alone should not contraindicate procedure when it is clinically beneficial [21].

Other guidelines [2,27] recommend delaying tracheostomy in COVID-19 patients until at least 14 days of intubation and suggest considering it in cases with stable pulmonary conditions, showing clinical improvement, and with a clear prognosis. Early pandemic studies [2,9,12], involving smaller patient numbers, suggested that FiO_2_ < 50%, PEEP<8, and P/F > 200 in 24 hours preceding surgery, and not requiring prone positioning, are important for a better postoperative prognosis. We were unable to achieve these values in most of patients.

These recommendations were also supported by Staibano’s study [28], which suggests that even when early tracheostomy is performed in critically ill patients, they may still be subjected to prolonged ventilatory support due to severe respiratory failure. Our cohort demonstrates that despite tracheostomy, the length of stay in ICU and hospital was still extended in patients who survived complications of viral pneumonia.

Currently, the timing of tracheostomy remains controversial. Considering the surgical timing in our cohort, most cases occurred around 2 weeks of intubation (median 16 days), consistent with literature [12,21]. Interestingly, while prolonged intubation remains classic surgical indication, there were a few cases of emergency tracheostomies [29] in our cohort (n = 3), an uncommon but notable occurrence.

### Additional considerations

Another aspect of tracheostomy in COVID-19 patients is potential risk of bleeding, given the disease’s nature, with coagulopathy, thrombosis, and frequent anticoagulant use [30]. We observed 9 cases of postoperative hemorrhage requiring reoperation, a higher rate than studies conducted before the pandemic [31] and similar to those in coronavirus series [8,29].

Although we did not specifically assess postoperative hypoxia, as observed by Martin-Villares [29], we encountered cases where the tracheostomy was non-functional despite the cannula being in place, leading to hypoxia and one intraoperative death.

Regarding location of procedure, Chiang’s meta-analysis [21] emphasizes the importance of a negative-pressure environment for tracheostomies in their cohort and suggests that if available in ICU, bedside surgery would be preferable to operating room, avoiding risk of transmission and complications during transport. However, operating room has advantages, such as better visualization, improved ergonomics for healthcare, and availability of equipment to manage complications. Mattioli [32] mentions that high-risk patients, such as obese individuals with short necks or enlarged thyroids, might benefit from transport to operating room. All of our tracheostomies were performed at bedside, individually and under negative pressure.

Concerning surgical technique, some studies advocate for open tracheostomy [12], while others favor percutaneous [22,23]. In reviewed studies, no difference in outcomes was found between techniques, and choice is recommended based on the surgeon’s familiarity and resource availability [21]. Our cohort only included open tracheostomy.

We achieved a high decannulation rate among surviving patients, higher than in other series [29]. Patients who did not decannulate had significant neurological deficits or airway stenosis confirmed by endoscopic examinations. Tornari [7] concluded that high FiO_2_ and increased cough flow before tracheostomy were related to a longer time to decannulation, which we did not assess. Similarly to our study, Tornari [7] also used progressively smaller cannula during removal process.

Considering that surgical complications were stable among all participants (deceased and survivors) it is important to note that high mortality was not directly related to procedure itself, but rather to severity of patients’ conditions. Our series had a high mortality rate, higher than others [29,33], but we did not find any studies with long data collection periods.

Finally, it is crucial to highlight importance of vaccination on mortality impact. Vaccination proved to be an independent and protective factor for outcome of COVID-19 tracheostomized patients. A comparison of characteristics before and after vaccine availability, with a cut-off date of 02/01/2021, is presented in the supplementary material table. While there were some subtle differences in median values for age and SAPS, as well as frequencies of amine use on the day of surgery and neoplasms in the first year of the pandemic, a uniformity of characteristics reinforces vaccination as a major protective factor influencing outcomes.

### Limitations

This study provides valuable real-world clinical data from a specialized infectious disease hospital in Brazil during the COVID-19 pandemic, based on a large sample size, offering insights into medical practice in a high-complexity setting. However, it is important to acknowledge its limitations, including its retrospective design, single-center setting, and reliance on medical records. Future research, including prospective studies and clinical trials, are necessary to validate these findings and develop evidence-based guidelines for optimizing tracheostomy management in critically ill COVID-19 patients. Such studies should focus on further exploring relationship between illness severity and outcomes, as well as effects of timing and other factors on survival.

### Future directions

Further research is warranted to confirm these findings and to explore the potential benefits of timely tracheostomy in this population, and their potential applicability to other diseases. Clinical trials evaluating optimized management strategies for COVID-19-related respiratory failure requiring tracheostomy are needed to develop evidence-based guidelines for improving patient care.

## Conclusions

This retrospective cohort study of 356 patients who underwent tracheostomy due to COVID-19-related respiratory failure at a referral infectious disease hospital in Brazil provides valuable insights into the multifactorial influences on mortality in this critically ill population.

We found that older age, pre-existing comorbidities such as chronic pulmonary disease, diabetes, renal injury, and lack of COVID-19 vaccination were associated with an increased risk of mortality. Admission severity scores did not reflect the patient’s condition at tracheostomy time and, therefore, had no correlation with outcomes. Additionally, performing a tracheostomy in context of hemodialysis and a low PaO2/FiO2 index, indicating poor oxygenation, may represent an unnecessary risk for patient.

Our analysis also reinforces the critical importance of disease severity at the time of tracheostomy, highlighting crucial need to assess both patient characteristics and clinical condition, rather than relying solely on duration of endotracheal intubation or mechanical ventilation. This approach could provide a valuable lesson to help reduce the high mortality of these patients in future pandemic outbreaks.

## Supporting information

S1 Table COVID-19 tracheostomy – Characteristics before and after vaccine availability (February 1, 2021). (DOCX)
