# Supplementary material for: Tracheostomy in COVID-19: A retrospective cohort study of outcomes and mortality predictors in a specialized infectious disease hospital in Brazil
Source: PLoS One. 2025 Jun 18;20(6):e0326531. doi: 10.1371/journal.pone.0326531 (PMC12176168; doi:10.1371/journal.pone.0326531)
Supplement: S1 Table — (DOCX) [file pone.0326531.s001.docx]

Title: Tracheostomy in COVID-19: A Retrospective Cohort Study of Outcomes and Mortality Predictors in a Specialized Infectious Disease Hospital in Brazil

**Supplementary Table:** **COVID-19 tracheostomy -** Characteristics before and after vaccine availability (February 1, 2021)

|  | **N** | **BEFORE** | **AFTER** | **Test Statistic** |  |
| --- | --- | --- | --- | --- | --- |
|  |  | **(N=118)** | **(N=238)** |  |  |
| Demographics and clinical characteristics | | | | | |
| Age, years (median, interquatile range) | 356 | 65 (54.6-73.9) | 61.8 (48.1-70.7) | p=**0.03**^2^ |  |
| Body mass index (interquatile range) | 356 | 27.8 (25.0-34.4) | 27.7 (24.8-32.0) | p=0.23^2^ |  |
| Cardiovascular disease | 356 | 4 (3.3) | 10 (4.2) | p=0.71^1^ |  |
| Chronic pneumopathy | 356 | 15 (12.7) | 29 (12.1) | p=0.89^1^ |  |
| Congestive heart disease | 356 | 7 (5.9) | 5 (2.1) | p=0.06^1^ |  |
| Diabetes | 356 | 38 (32.2) | 58 (24.3) | p=0.12^1^ |  |
| Hemodialysis (previous) | 356 | 1 (0.8) | 3 (1.2) | p=0.73^1^ |  |
| HIV/AIDS | 356 | 5 (4.2) | 17 (7.1) | p=0.28^1^ |  |
| Hypertension | 356 | 77 (0.6) | 136 (57.1) | p=0.14^1^ |  |
| Neoplastic disease | 356 | 5 (4.2) | 2 (0.8) | p=**0.03**^1^ |  |
| Tuberculosis | 356 | 1 (0.8) | 12 (5.0) | p=0.05^1^ |  |
| Oxygen delivery/therapy device at arrival | 356 |  |  | p=**0.03**^1^ |  |
| *Room air* |  | 13 (11.0) | 31 (13.0) |  |  |
| *Conventional oxygen therapy* |  | 63 (53.3) | 143 (60.0) |  |  |
| *Invasive endotracheal intubation* |  | 42 (35.5) | 64 (26.8) |  |  |
| SOFA, points (median, interquatile range) | 356 | 4.5 (2.0-8.0) | 4 (2-7) | p=0.43^2^ |  |
| SAPS 3, points (median, interquatile range) | 356 | 54.0 (46.9-72.2) | 52 (43-67) | p=**0.03**^2^ |  |
| Sex at birth (male) | 356 | 67 (56.7) | 145 (60.9) | p=0.45^1^ |  |
| Time of disease at admission, days (median, interquatile range) | 356 | 5.5 (3-9) | 7 (4-9.1) | p=0.08^2^ |  |
| Vaccines doses (COVID-19, any, interquatile range) | 305 | 0.0 (0.0-0.0) | 1.0 (0.0-2.0) | p=0.01^2^ |  |
| Severity of illness at the time of tracheostomy and surgical complications | | | | | |
| Amine use on surgery day | 356 | 77 (65.2) | 126 (52.9) | p=**0.03**^1^ |  |
| Coagulation issue on surgery day^a^ | 356 | 7 (5.9) | 26 (10.9) | p=0.13^1^ |  |
| Hemodialysis on surgery day | 356 | 40 (3.3) | 81 (34.0) | p=0.98^1^ |  |
| P/F on surgery day (interquatile range) | 356 | 263 (185-365.8) | 260.5 (187.8-396.3) | p=0.95^2^ |  |
| Postoperative complications | 356 | 9 (7.6) | 16 (6.7) | p=0.75^1^ |  |
| Surgical duration ≥ 40 min | 356 | 9 (7.6) | 11 (4.6) | p=0.25^1^ |  |
| Technical difficulties during surgery | 356 | 16 (13.5) | 25 (10.5) | p=0.40^1^ |  |
| Outcomes | | | | | |
| Decannulated patients | 356 | 38 (32.2) | 67 (28.1) | p=0.43^1^ |  |
| Duration of MV, days (median, interquatile range) | 356 | 31 (23.9-42.1) | 27.5 (22-41) | p=0.10^2^ |  |
| Duration of MV before TCT, days (median, interquatile range) | 356 | 16 (14-19.1) | 17 (14-19) | p=0.88^2^ |  |
| Hospital mortality | 356 | 79 (66.9) | 163 (68.4) | p=0.77^1^ |  |
| ICU mortality | 356 | 74 (62.7) | 160 (67.2) | p=0.40^1^ |  |
| LOS before INI, days (median, interquatile range) | 356 | 2 (1 - 4) | 2 (1 - 3.1) | p=0.38^2^ |  |
| LOS INI before ICU, days (median, interquatile range) | 356 | 0.0 (0.0-0.0) | 0.0 (0.0-0.0) | p=0.18^2^ |  |
| LOS INI ICU, days (median, interquatile range) | 356 | 335 (25-51.1) | 33 (25-47) | p=0.50^2^ |  |
| LOS INI, days (median, interquatile range) | 356 | 36.5 (27-63.1) | 38 (26-56.1) | p=0.70^2^ |  |
| Weaning from MV | 356 | 40 (3.3) | 69 (28.9) | p=0.34^1^ |  |

**Bold: p-value <0.05**. ^1^Pearson. ^2^Wilcoxon. ^a^Coagulation issue was defined as: thrombocytopenia (<100,000), or prolonged Prothrombin Time (PT) and/or activated Partial Thromboplastin Time (aPTT); or active bleeding and/or the need for plasma and/or platelet transfusions for the surgical procedure. Cardiovascular disease: 11 cases of myocardial infarction, 2 cases of cerebrovascular disease and 1 case of peripheral arterial disease. Chronic pneumopathy: GOLD III/IV chronic obstructive pulmonary disease. COVID-19: Coronavirus disease 2019 by SARS-CoV-2; Congestive heart disease: heart functional classification by New York Heart Association - NYHA ≥II. Hemodialysis (previous): end-stage renal disease on hemodialysis before admission; HIV/AIDS: human immunodeficiency virus/acquired immunodeficiency syndrome; ICU: intensive care unit; INI: Instituto Nacional de Infectologia; LOS: length of stay; MV: mechanical ventilation; Neoplastic disease: 5 cases of solid tumors and 2 cases of hematologic neoplasms undergoing oncological treatment.; P/F: the ratio of arterial oxygen partial pressure to fractional inspired oxygen; SAPS (Simplified Acute Physiology Score) 3; SOFA: sequential organ failure assessment score. STE: standard error; TCT: tracheostomy.
